# Population genetic structure of *Plasmodium falciparum* across a region of diverse endemicity in West Africa

**DOI:** 10.1186/1475-2875-11-223

**Published:** 2012-07-03

**Authors:** Victor A Mobegi, Kovana M Loua, Ambroise D Ahouidi, Judith Satoguina, Davis C Nwakanma, Alfred Amambua-Ngwa, David J Conway

**Affiliations:** 1 Department of Pathogen Molecular Biology, London School of Hygiene and Tropical Medicine, London, WC1E 7HT, UK; 2 Medical Research Council Unit, Fajara, Banjul, The Gambia; 3 National Institute of Public Health, Conakry, Republic of Guinea; 4 Université Cheikh Anta Diop, Dakar, Senegal

**Keywords:** *Plasmodium falciparum*, Microsatellite, Population structure, Transmission intensity

## Abstract

**Background:**

Malaria parasite population genetic structure varies among areas of differing endemicity, but this has not been systematically studied across *Plasmodium falciparum* populations in Africa where most infections occur.

**Methods:**

Ten polymorphic *P. falciparum* microsatellite loci were genotyped in 268 infections from eight locations in four West African countries (Republic of Guinea, Guinea Bissau, The Gambia and Senegal), spanning a highly endemic forested region in the south to a low endemic Sahelian region in the north. Analysis was performed on proportions of mixed genotype infections, genotypic diversity among isolates, multilocus standardized index of association, and inter-population differentiation.

**Results:**

Each location had similar levels of pairwise genotypic diversity among isolates, although there were many more mixed parasite genotype infections in the south. Apart from a few isolates that were virtually identical, the multilocus index of association was not significant in any population. Genetic differentiation between populations was low (most pairwise *F*_ST_ values < 0.03), and an overall test for isolation by distance was not significant.

**Conclusions:**

Although proportions of mixed genotype infections varied with endemicity as expected, population genetic structure was similar across the diverse sites. Very substantial reduction in transmission would be needed to cause fragmented or epidemic sub-structure in this region.

## Background

*Plasmodium falciparum* causes an annual burden of hundreds of millions of episodes of clinical malaria, and between approximately 0.5 and 1.5 million deaths, mostly in endemic populations of sub-Saharan Africa [1-3]. Transmission intensity of malaria parasites is highly variable temporally and geographically [4,5], and this variation plays a role in determining parasite population genetics and evolution locally. When human malaria infections contain a mixture of multiple haploid parasite clones, genetically different gametocytes may be taken into a mosquito blood meal, leading to heterozygous diploid parasites and meiotic reassortment and recombination as the haploid products are formed [6]. However, when infections are uncommon and mostly occur as single genotypes, there would be relatively more inbreeding of the parasites [7], leading to likely changes in population genetic structure during malaria control and elimination.

Microsatellite surveys of *P. falciparum* from endemic populations have clearly shown that infections are less genotypically mixed in areas of low transmission, which does apparently reduce the effective recombination rate and allow linkage disequilibrium to persist locally [8]. Such a pattern occurs in many populations in South America and Southeast Asia, and a wide spectrum of population structures can be seen among different sites within individual countries such as Brazil [9], Malaysia [10], Thailand [8,11,12], Philippines [13], and Papua New Guinea [8,14]. The degree of isolation and genetic differentiation of local subpopulations within countries also varies in these areas, due to differences in the history of discrete transmission foci as well as some fragmentation in the distribution of current endemicity [8-14].

In contrast to the situation in such regions, most of the global *P. falciparum* infections occur in sub-Saharan Africa, and there is continuous endemicity throughout most of this region [1,5]. A number of independent studies indicate that local *P. falciparum* populations in Africa are genetically very diverse, with a high effective recombination rate due to many mixed genotype infections [6,8,15]. Furthermore, local populations are not strongly isolated from each other due to frequent migration of humans within sub-regions of the continent [8,16,17]. Exceptions are populations on the very edge of the endemic distribution such as in Djibouti [18], or on remote islands such as the Comoros [19], that show a fragmented structure as expected. However, individual studies have tended to focus on only one or a few sites, and parasite population structures in Africa have not been compared very systematically. It is particularly important to do so at this time, as substantial reductions in malaria in parts of Africa have recently occurred [20]. Such changes may have started to affect parasite population genetics, an understanding of which is needed for considering potential elimination or sustained endemic control.

Here, the genetic structure of *P. falciparum* populations was compared among eight locations sampled in four contiguous countries in West Africa (Senegal, Gambia, Guinea Bissau, and Republic of Guinea). These sites vary in endemicity on a north–south gradient of seasonal rainfall which correlates with transmission [5], with ecology ranging from Sahel in the north to dense savannah and rainforest in the south. In the lower endemic northern areas there has recently been more malaria control, particularly in The Gambia and Senegal where significant decline in malaria has been observed during the past decade [21-23]. A set of ten highly polymorphic microsatellite loci was used to determine proportions of mixed genotype infections and pairwise differences among genotypic profiles of isolates, and to assess multi-locus allelic associations within populations and the degree of genetic differentiation among populations. The findings have implications for understanding the epidemiology of malaria in this region, and the potential for more effective control.

## Methods

### *Plasmodium falciparum* DNA samples

**Figure 1:**
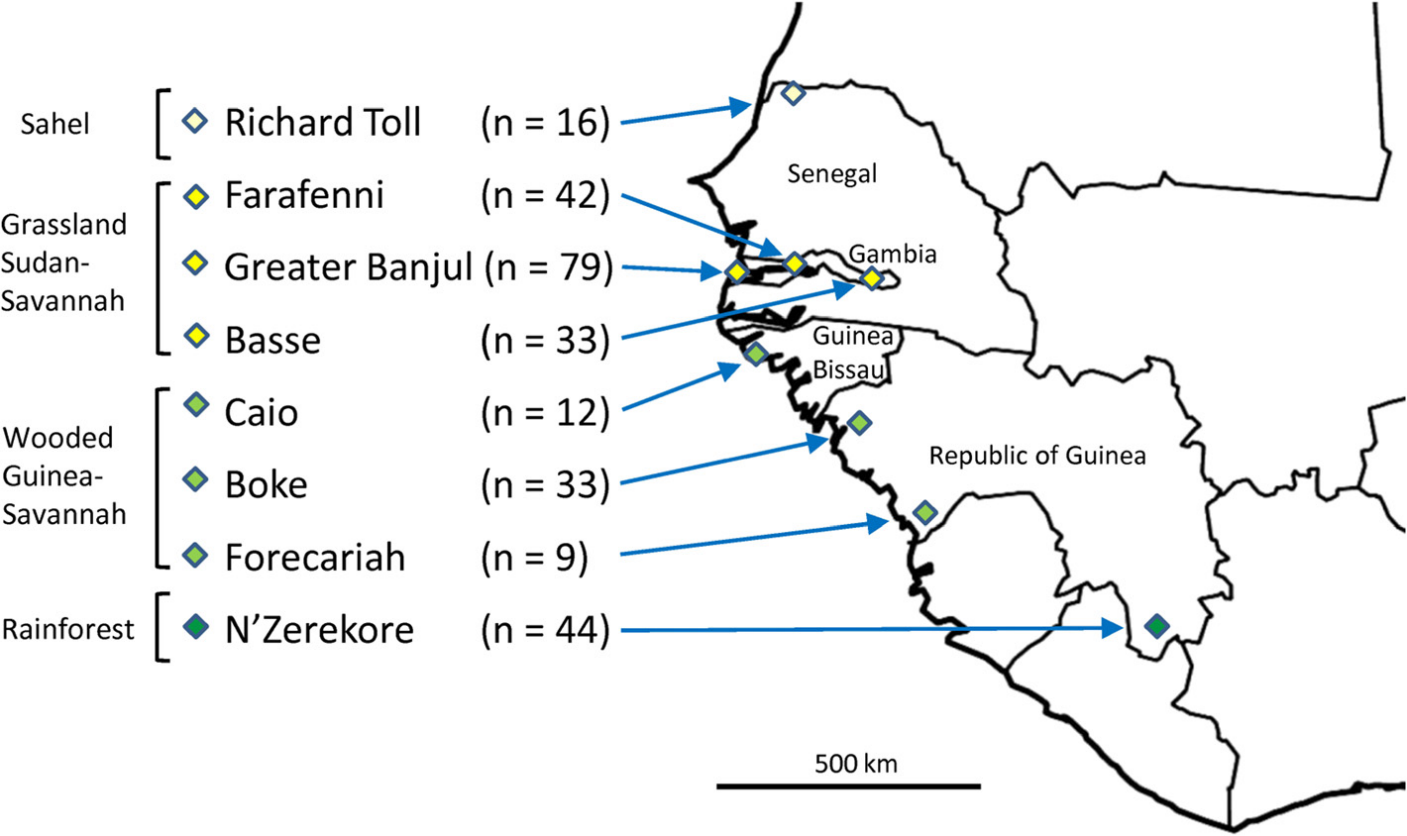
**Location of 8 West African sites sampled for study of*P. falciparum*population structure, with analysis of 10 microsatellite loci in a total of 268 isolates (sample sizes at each of the sites are given in parentheses).** In this region, transmission shows a gradient from low levels in the north to higher levels in the south [5].

Genotyping of *P. falciparum* was performed on DNA extracted from blood samples from 268 infected individuals, sampled from eight sites in four West African countries between 2005 and 2009 (Figure 1). All subjects or their guardians gave written informed consent to provide a blood sample for studies of malaria that included genotyping of malaria parasites. Protocols were reviewed and approved by the Gambia Government and MRC joint Ethics Committee and the Ethics Committee of the London School of Hygiene and Topical Medicine, and investigators adhered to current guidelines on Good Clinical Practice. For five of the sites, in The Gambia (Basse, Farafenni and Greater Banjul area), Senegal (Richard Toll), and Guinea Bissau (Caio), DNA was previously extracted from blood samples positive for *P. falciparum* by slide examination, and those also positive by PCR with sufficient material for genotyping were analyzed here. The samples from Basse (n = 33), Farafenni (n = 42) and Caio (n = 12) were from community surveys conducted in January and February 2008 [24], shortly after the end of the 2007 malaria transmission season (most cases occur between August and November each year). Samples from the Greater Banjul area (n = 79) were from clinical cases positive for *P. falciparum* by microscopy presenting to four local health facilities (Royal Victoria Teaching Hospital in Banjul, the MRC clinic in Fajara, Jammeh Foundation for Peace Hospital in Serekunda, and Brikama Health Centre) during the malaria seasons of 2005–2009 [25]. Samples from Richard Toll in northern Senegal (n = 16) were collected from the Centre de Santé de Richard Toll during the malaria season of 2005 and were determined to be *P. falciparum* positive in a survey for parasite detection and drug resistance. In the Republic of Guinea, 105 filter-paper blood samples were collected from patients presenting with malaria between December 2009 and February 2010 who were positive for malaria parasites by rapid diagnostic test (RDT) and slide examination at the Government Health Centres in Boke, N’Zerekore and Forecariah. Genomic DNA was extracted in The Gambia using a Corbett Robotics X-Tractor Gene^TM^ robot (Corbett Robotics Pty Ltd, Australia), and *Plasmodium* species identification was performed on the DNA samples using genus-and species-specific primers [26], identifying 102 *P. falciparum*-positive samples from Guinea, of which 86 had sufficient DNA for multi-locus microsatellite analysis here (44 from N’Zerekore, 33 from Boke, and 9 from Forecariah).

### Microsatellite genotyping of *Plasmodium falciparum*

*Plasmodium falciparum* DNA from each individual sample was genotyped for 10 polymorphic microsatellite marker loci, chosen from a set of 12 previously described [27] (two loci that gave less efficient PCR amplification were not included). These loci consist of specific sequences flanking (TAA)_n_ repeats in the haploid *P. falciparum* genome [28], and were PCR-amplified using a heminested protocol [27] with slight changes in PCR reaction mix constituent concentrations and primer dye labels: 6-FAM for locus *TA1* on chromosome 6 (Chr 6), *TA81* (Chr 5) and *ARA2* (Chr 11); VIC for *TA42* (Chr 5) and *PfPK2* (Chr 12); PET for *POLYα* (Chr 4), *TA87* (Chr 6) and *TA60* (Chr 13); NED for *TA109* (Chr 6) and *Pfg377* (Chr 12).

PCR products for the different loci amplified from each individual DNA sample were pooled into two groups of 5 loci together with GeneScan™ 500 LIZ internal size standard (Applied Biosystems, Foster City, CA) for electrophoresis on an ABI 3130XL Genetic Analyzer. One pool comprised loci *TA42*, *TA109*, *POLYα*, *TA60* and *TA81* while the other comprised *TA1*, *TA87*, *ARA2*, *Pfg377* and *PfPK2* for a given individual sample. GeneMapper software version 4 (Applied Biosystems, UK) was used for scoring the allele sizes and for quantifying peak heights for samples containing multiple alleles per locus. Multiple alleles per locus were scored if electrophoretic peaks corresponding to minor alleles were ≥20% the height of that for the predominant allele in the isolate.

### Microsatellite data analysis

For each locus in each individual isolate, the predominant allele (where multiple alleles were detected) or the only allele (where only a single allele was scored) was counted for population genetic analyses. Summary indices including numbers of alleles, allelic diversity within each population, and allele frequencies per locus per population were calculated using FSTAT Version 2.9.3.2 (updated from [29]). Allelic diversity was calculated for each of the 10 microsatellite loci based on the allele frequencies, using the formula for ‘expected heterozygosity’ *H*_*e*_ = [n/(n − 1)][1 − *∑p*^2^, where n is the number of isolates analyzed and *p* represents the frequency of each different allele at a locus. *H*_*e*_ has a potential range from 0 (no allele diversity) to 1 (all sampled alleles are different).

For isolates that were fully genotyped at all 10 loci, analysis of multi-locus genotypic profiles of the isolate was performed, considering the majority allele at each locus in the case of mixed genotype infections. Pairwise comparisons among all isolates within each population were performed using Splitstree [30] to generate and graphically visualize a matrix of similarity among isolates based on numbers of identical or mismatched loci. To test for existence of multilocus linkage disequilibrium, the standardized index of association (*I*_*A*_^*S*^) was calculated, based on genotypic profiles of the majority allele at each locus in each infection, using the LIAN version 3.5 Web interface [31]. This index is calculated as *I*_*A*_^*S*^ = 1/L-1(V_D_/ V_E_-1), where L is the number of loci used, and the variance (V_D_) in pairwise numbers of mismatched alleles between isolates was compared with that expected under linkage equilibrium (V_E_) by simulation of data by 10000 iterations under the null hypothesis of V_D_ = V_E_ by Monte Carlo random sampling [31].

The level of genetic differentiation between the populations was calculated using fixation indices (*F*_ST_) computed with FSTAT, and a test for isolation by distance was performed from the pairwise genetic distances and geographical distances between populations, using a Mantel test of matrix correlation (Genepop version 4.0.10 web interface, Isolde program) [32].

## Results

All of the ten microsatellite loci were highly polymorphic in all eight sites from the four countries sampled, with the overall numbers of different alleles per locus ranging from 7 (for locus *TA42*) to 24 (for locus *Polyα*). For each of the 268 isolates, the genotype profile is listed in Additional file 1, and allele frequencies at each of the ten loci in each of the eight sites are given in Additional file 2. All isolates were genotyped successfully for at least eight of the ten loci, and 186 (69.4%) isolates had results for all loci (most missing data were from locus *TA42* which was genotyped in 76.9% of isolates) ( Additional file 1). Allelic diversity at each locus was summarized as the expected heterozygosity (*H*_*e*_) from the distribution of allele frequencies, and was similar across all the sites, with averaged values across the loci between 0.72 (for Richard Toll) and 0.80 (for Caio) (Table 1; P > 0.1 for each Wilcoxon Signed Ranks test on pairs of sites).

**Table 1:** **Allelic diversity (expected heterozygosity,*H***_**e**_**) at 10 microsatellite loci in 8 local populations of*Plasmodium falciparum*in West Africa**

	**Populations sampled (n = numbers of individual isolates)**							
		**Guinea**		**Guinea Bissau**		**Gambia**		**Senegal**
**Locus**	**N’Zerekore (n = 44)**	**Boke (n = 33)**	**Forecariah (n = 9)**	**Caio (n = 12)**	**Basse (n = 33)**	**Farafenni (n = 42)**	**Greater Banjul (n = 79)**	**Richard Toll (n = 16)**
*TA1*	0.85	0.88	0.89	0.91	0.88	0.85	0.86	0.91
*TAA87*	0.85	0.79	0.81	0.82	0.86	0.85	0.85	0.74
*ARA2*	0.88	0.88	0.72	0.85	0.84	0.82	0.82	0.71
*Pfg377*	0.64	0.57	0.56	0.64	0.63	0.52	0.53	0.69
*PfPK2*	0.87	0.85	0.86	0.89	0.88	0.89	0.85	0.93
*Polya*	0.85	0.90	0.83	0.96	0.78	0.89	0.89	0.86
*TAA60*	0.80	0.83	0.89	0.68	0.82	0.79	0.82	0.75
*TAA81*	0.85	0.83	0.89	0.74	0.84	0.80	0.79	0.76
*TAA109*	0.83	0.86	0.86	0.85	0.88	0.86	0.87	0.82
*TA42*	0.34	0.54	0.39	0.68	0.16	0.22	0.25	0.00
Mean	0.78	0.79	0.77	0.80	0.76	0.75	0.75	0.72

The number of genotypes detected in an isolate was defined as the maximum number of alleles scored at any of the individual loci. The numbers of parasite genotypes detected per isolate differed substantially across populations (Kruskal-Wallis test, P < 0.001), and tended to be higher at sites in the south (Figure 1 and Table 2). In particular, the three sites in the Republic of Guinea had significantly higher numbers (means of 4.2 in Forecariah, 4.1 in Boke, and 3.7 in N’Zerekore) than each of the other sites, in Guinea-Bissau (mean of 2.6 in Caio), Senegal (2.2 in Richard Toll) and The Gambia (2.4 in Basse, 2.1 in Farafenni and 1.7 in the Greater Banjul area) (Table 2; P < 0.001 for each Mann–Whitney test). Remaining pairwise comparisons among sites were not significant, except that the numbers of genotypes per isolate were significantly lower in the Greater Banjul area than in each of the other sites (P < 0.005) except for Richard Toll. In a complementary analysis, the proportion of individual locus scores with more than one allele per isolate was highest in Nzerekore (0.58) and lowest in the Greater Banjul area (0.14) (Table 2 and Additional file 1).

**Table 2:** **Multiple genotype infections assessed by typing 10*P. falciparum*microsatellite loci in 268 isolates from 8 West African locations**

**Country**	**Location**	**Number of isolates**	**Number of isolates with given no. of detected genotypes**					**Mean number of genotypes per isolate**^**a**^	**Proportion of locus scores with >1 allele**^**b**^
			**1**	**2**	**3**	**4**	**>4**		
Guinea	N’Zerekore	44	1	6	8	14	15	3.7	0.58
Guinea	Boke	33	0	3	5	14	11	4.1	0.54
Guinea	Forecariah	9	0	0	2	3	4	4.2	0.51
Guinea Bissau	Caio	12	0	6	5	1	0	2.6	0.30
Gambia	Greater Banjul	79	34	32	13	0	0	1.7	0.14
Gambia	Basse	33	1	20	11	1	0	2.4	0.35
Gambia	Farafenni	42	6	26	8	2	0	2.1	0.33
Senegal	Richard Toll	16	3	9	3	0	1	2.2	0.26

^a^For this analysis the number of genotypes was scored from the locus with the largest number of alleles within the isolate.

^b^For this analysis the proportion of loci with >1 allele within each isolate was scored.

**Figure 2:**
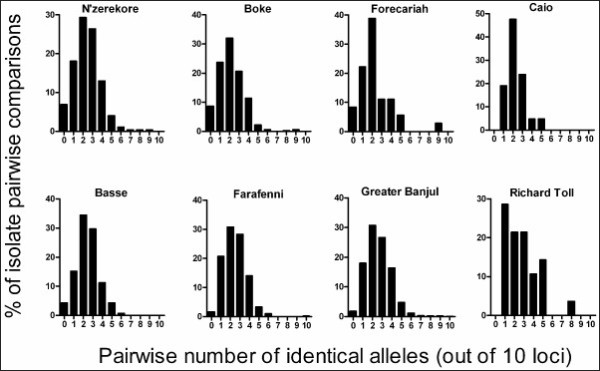
**Distribution of similarity indices of the 10 locus genotype profiles among all pairs of isolates within each of the sampled populations.** Median similarity in each population is only 2 out of 10 loci with matching alleles. Hardly any isolates match at more than 5 loci, except for very few pairs that are virtually identical.

Most isolates had complete genotype data for all loci, and in these the multilocus profiles were examined, considering the majority allele at each of the ten loci. Almost all isolates differed from each other at most loci, and for each population the median number of loci with matching alleles in pairwise comparisons was only two out of 10 (Figure 2). However, a very small number of isolates showed exceptionally high level of identity, with six of the eight populations containing at least one pair of isolates matching at 8 or more loci (matching genotypes were different in each population). For example, in Farafenni a single pair of isolates were identical at all loci while none of the other pairs were identical at more than 6 loci, and in Richard Toll one pair of isolates were identical at 8 loci while none of the rest were identical at more than 5 loci (Figure 2). Performing an initial multi-locus index of association (*I*_*A*_^*S*^) test, significant values were detected in five of the populations (Table 3), but after removal of individual isolates matching another at 8 or more loci none of the *I*_*A*_^*S*^ values was significant (Table 3). As many isolates had mixed genotypes, the dominant multilocus profile in some could be a composite of different genotypes rather than a true haplotype, but previous analysis of other populations shows very similar *I*_*A*_^*S*^ results whether only including single genotype infections or a more broad inclusion of profiles from all infections [8]. Here, there was a sufficient number of single-clone isolates fully genotyped in the Greater Banjul area for a separate analysis (n = 26), which also showed no significant index of association (*I*_*A*_^*S*^ = 0.020, P > 0.05).

**Table 3:** **Standardized multi-locus Index of Association (*I***_***A***_^***S***^**) test for*Plasmodium falciparum*isolates genotyped for a complete set of 10 microsatellite loci at 8 West African locations**

**Country**	**Location**	**Including all isolates**			**Non-identical genotypes**^§^		
		**n**	***I***_***A***_^***S***^		**n**	***I***_***A***_^***S***^	
Guinea	N’Zerekore	24	0.036	**	22	0.019	^NS^
Guinea	Boke	26	0.032	**	23	0.011	^NS^
Guinea	Forecariah	9	0.106	***	8	0.041	^NS^
Guinea Bissau	Caio	7	0.000	^NS^	7	0.000	^NS^
Gambia	Basse	24	0.008	^NS^	24	0.008	^NS^
Gambia	Farafenni	25	0.007	^NS^	24	0.000	^NS^
Gambia	Greater Banjul	63	0.017	**	59	0.009	^NS^
Senegal	Richard Toll	8	0.150	***	7	0.061	^NS^

n = number of isolates analysed; ^§^ to exclude genotypes that were virtually identical one member of each isolate pair matching at 8 or more of the 10 loci was removed (2 isolates in N’Zerekore, 3 in Boke; 1 in Forecariah, 1 in Farafenni, 4 in Greater Banjul and 1 in Richard Toll); ^NS^ P > 0.05, not significant; * *P* < 0.05; ** *P* < 0.01; *** *P* < 0.001.

**Figure 3:**
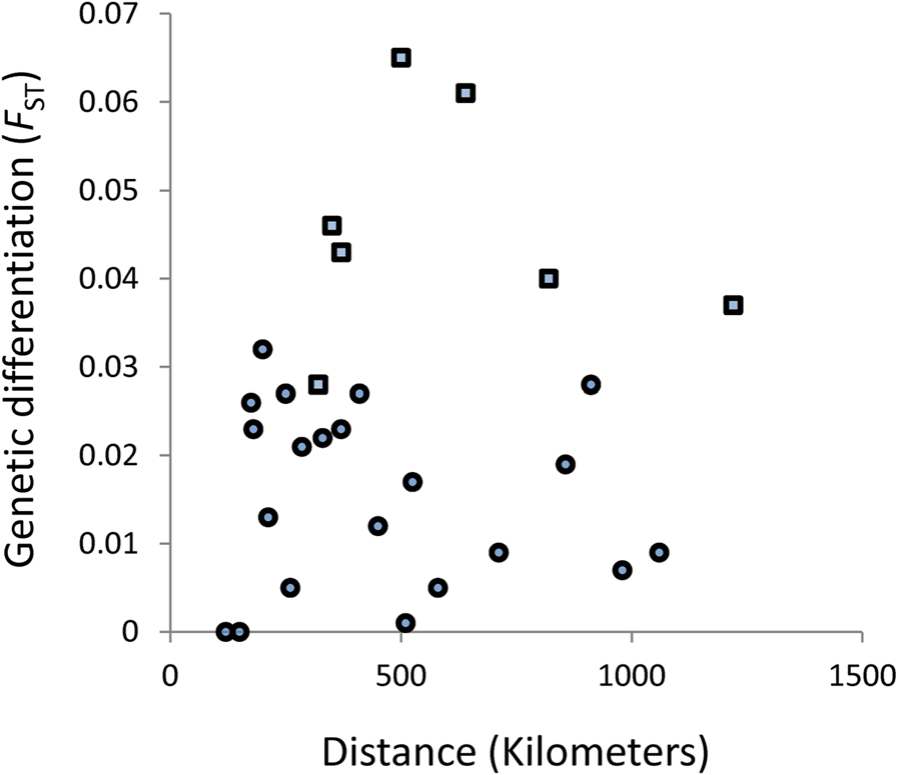
**Scatterplot of pairwise genetic divergence between populations (*F***_**ST**_**mean for all 10 microsatellite loci) plotted by geographical distance in kilometers between them.** Comparisons that include the northernmost population (Richard Toll) are shown with square points, while all others are shown with circular points. An overall test for isolation by distance was not significant (Mantel test of matrix correlation, P = 0.304).

Most alleles were distributed widely across different populations, and any ‘private’ alleles (detected only in one population) were at very low frequencies ( Additional file 2). Pairwise comparisons of populations showed that allele frequencies were similar and the *F*_ST_ values were low, ranging from not significantly greater than zero for many population pairs, through to *F*_ST_ = 0.065 (P = 0.048) between Richard Toll and Caio (Table 4). The *F*_ST_ values and geographical distances between all pairs of populations were analysed by a Mantel test of matrix correlation, showing no significant overall evidence for isolation by distance (P = 0.304). However, the northernmost site at Richard Toll tended to be more divergent from the rest of the populations (with *F*_ST_ values of 0.028 to 0.065; Figure 3 and Table 4).

**Table 4:** **Matrix of genetic differentiation (*F***_**ST**_**values below diagonal) and geographical distances (in Km above diagonal) in pairwise comparisons of eight sampled*P. falciparum*populations**

	**Nzr**	**Bok**	**For**	**Bas**	**Cai**	**Far**	**Gba**	**Ric**
Nzr		696	504	852	932	980	1055	1218
Bok	0.009		213	262	235	323	372	629
For	0.001	0.013		446	439	533	582	825
Bas	0.019	0.021	0.012		264	151	252	382
Cai	0.028	0.032	0.027	0.027		193	174	504
Far	0.007	0.022	0.017	0.000	0.023		104	320
Gba	0.009	0.023	0.005	0.005	0.026	0.000		346
Ric	0.037	0.061	0.040	0.043	0.065	0.028	0.046	

Nzr = N’Zerekore; Bok = Boke; For = Forecariah; Bas = Basse; Cai = Caio; Far = Farafenni; Gba = Greater Banjul area; Ric = Richard Toll.

Pairwise *F*_ST_ values are the mean for all 10 microsatellite loci, and values significantly above zero (p < 0.01) are underlined. Sample sizes for each location are given in Methods and Table 1.

## Discussion

Across a marked gradient in transmission intensity among West African locations sampled here, the parasite populations show remarkably similar population genetic structure. There was a high allelic diversity for the ten *P. falciparum* microsatellite loci in all the eight locations, the overall *H*_*e*_ index for each site ranging from 0.72 to 0.80, the same range as that previously seen in most other endemic African countries [8,16,33]. Although slightly lower diversity has been reported in a low-endemic area of Dakar in Senegal [18] and a highland area of western Kenya [34], a much lower diversity has only been reported for one African location (*H*_*e*_ of 0.41 in Djibouti which has unstable epidemic transmission) [18].

As expected, the mean numbers of *P. falciparum* genotypes detected per infection varied among the populations studied, with highly mixed infections at each of the sites in Guinea which experience high transmission for much of the year, in contrast with sites having limited seasonal transmission further north in the region. This substantial difference is likely to be due to differences in endemicity as predicted, rather than confounding due to differences in sampling. Samples from five of the sites were from clinical cases presenting to health facilities, and those from the remaining three sites were from community sampling of asymptomatic individuals, but the latter sites were in the middle of the range geographically and in terms of proportions of mixed genotype infections, so this did not cause the observed north–south contrast in values. Review of comparisons elsewhere between asymptomatic and symptomatic infections also shows no consistent overall difference in proportions of mixed genotype infections [35-40].

The multi-locus index of association analysis initially indicated non-random patterns in several populations, but this was shown to be entirely due to only one or a few pairs of virtually identical isolates. In South American and Southeast Asian populations of low endemicity, identical *P. falciparum* isolates are sometimes seen as multi-locus genotypes persist through a number of self-fertilisation and transmission cycles. This has allowed comparisons of identical versus non-identical pairs of clones for estimation of phenotype heritability in a manner analogous to a conventional twin-pair study [41,42]. In Africa, related parasites may occur within isolates but it is rare to see identical genotypes in different infections [43]. It has been previously shown in The Gambia that identical parasite genotypes were more common in pairs of children sleeping in the same house who presented with malaria on the same day [44], and occasionally in children living close to each other within small villages [45]. Such identical genotypes probably result from single mosquitoes infecting more than one individual or from transmission of parasites by different mosquitoes that fed on a single genotype gametocyte carrier.

As was seen here, it has elsewhere been shown that inclusion of a very small number of closely related parasite genotypes can generate a significantly non-random multi-locus index of association, even when there is no linkage disequilibrium among the loci in the population generally [8]. Such occasional occurrence of identical parasites should not lead to a population being considered ‘clonal’, as such a term is not generally applied to human population genetic structure despite the presence of monozygotic twins. Instead, it has been suggested that the presence of many pairs of genotypically similar isolates may be taken as evidence of an ‘epidemic’ population structure [8,46], but here there were very few such pairs and none of the populations could be described as having such a structure. It should be noted that if multiple sibling parasite genotypes within the same host were included in a crude analysis, it could give false appearance of linkage disequilibrium [47,48]. Therefore, such a problem is avoided by restricting analysis to one parasite genotype per host [49-51], following the general principle whereby genotypes from closely related family members are not separately counted in studies of human linkage disequilibrium.

Over the sampled range of up to ~ 1200 kilometers between sites in West Africa, this microsatellite analysis found very low levels of genetic differentiation between the local populations of *P. falciparum*, with most pairwise *F*_ST_ values being less than 0.03. This is considerably less than the differentiation among local sites within non-African countries that are less endemic, including Malaysia [10], Papua New Guinea [14], the Philippines [13], and Brazil [9], with *F*_ST_ values exceeding 0.10 between sites separated by similar distances. It is instead consistent with previous sampling from more widely separated African populations, which has shown *F*_ST_ values of less than 0.05 for a similar set of microsatellite loci [8,16]. Despite the ecological and epidemiological diversity in West Africa, there is likely to be considerable mixing of parasites between different locations, due to frequent movement of humans in this region [17]. In comparison with South America and Southeast Asia, it will be difficult to identify discrete endemic locations in West Africa where malaria elimination might be achieved and sustained in the face of local migration.

Despite studying only ten polymorphic loci and sampling a very limited number of isolates from some of the sites, marked variation in proportions of multiple clone infections and relatively similar genetic structure of *P. falciparum* populations has been clearly shown, providing a framework for future genomic-scale studies. Comprehensive analysis of genome sequence variation should allow finer differences in population structure to be detected [52], including variation in patterns across the genome and identification of genes under natural selection, particularly with large sample sizes. Given the high levels of recombination and minimal reproductive isolation of parasite populations in West Africa, we predict that differential signatures of selection in particular populations will be detectable against a background of neutral genomic variation that is more spatially homogeneous.

## Conclusions

This analysis of ten microsatellite loci in *P. falciparum* in West Africa showed there were more mixed parasite genotype infections in highly endemic forested areas in the south than in drier areas with lower transmission in the north, although each location showed similar levels of allelic diversity and pairwise genotypic diversity among isolates. Apart from a few exceptional pairs of isolates that were virtually identical, there was no significant multilocus index of association in any population, genetic differentiation between locations was low, and an overall test for isolation by distance was not significant. Substantial future reduction in transmission would be needed before fragmented or epidemic sub-structure in parasite populations is likely to be seen in this region. Given the high levels of recombination and minimal reproductive isolation of parasite populations in this region, genome-wide studies may detect differential signatures of selection against a background of neutral variation that is relatively spatially homogeneous.

## Competing interests

The authors declare that they have no competing interests.

## Authors’ contributions

VAM and DJC designed the experiments. VAM performed the experiments. VAM and DJC analysed the data. VAM, KML, ADA, JS, DCN, AA-N, and DJC provided materials and tools. VAM and DJC wrote the paper. All authors read and approved the final manuscript.

## Supplementary Material

Additional file 1 **Table S1. Alleles scored in each of the 268*P. falciparum*isolates genotyped at 10 microsatellite loci.** Microsatellite alleles detected in isolates from the eight sampled populations, with highlighting showing predominant allele calls in the mixed genotypes. Click here for file

Additional file 2 **Table S2. Allele frequencies at 10 microsatellite loci in each of the eight population samples of*Plasmodium falciparum.*** Data on frequency of each allele scored for each microsatellite locus in each of the eight populations sampled. Click here for file
