# Supplementary material for: Population genetic structure of Plasmodium falciparum across a region of diverse endemicity in West Africa
Source: Malar J. 2012 Jul 3;11:223. doi: 10.1186/1475-2875-11-223 (PMC3425276; doi:10.1186/1475-2875-11-223)
Supplement: Additional file 1 — Table S1. Alleles scored in each of the 268P. falciparumisolates genotyped at 10 microsatellite loci. Microsatellite alleles detected in isolates from the eight sampled populations, with highlighting showing predominant allele calls in the mixed genotypes. [file 1475-2875-11-223-S1.docx]

**Table S1.** Alleles scored in each of the 268 *P. falciparum* isolates genotyped at 10 microsatellite loci

| S.No | Sample ID | TA1 | TAA87 | ARA2 | PF377 | PFPK2 | Polyα | TAA60 | TAA81 | TAA109 | TA42 |
| --- | --- | --- | --- | --- | --- | --- | --- | --- | --- | --- | --- |
| 1 | NZR1 | 160 | 105 | 68 | 100 | 163 | 159 |  | 116 | 163 | 201 |
| 2 | NZR2 | 166 | 105 | 62 | 94 | 181 | 168 | 84 | 152 | 175 | 186 |
| 3 | NZR4 | 169 | 99 | 65 | 94 | 169 | 162 |  | 110 | 178 | 186 |
| 4 | NZR5 | 169 | 96 | 95 | 97 | 169 | 156 | 84 | 119 | 163 | 186 |
| 5 | NZR6 | 163 | 102 | 74 | 100 | 157 | 138 | 75 | 119 | 163 | 186 |
| 6 | NZR7 | 166 | 102 | 68 | 100 | 163 | 156 | 84 | 152 | 178 | 186 |
| 7 | NZR8 | 160 | 105 | 98 | 100 | 160 | 168 | 87 | 122 | 163 | 186 |
| 8 | NZR9 | 163 | 99 | 98 | 100 | 160 | 159 |  | 116 |  | 186 |
| 9 | NZR10 | 172 | 96 | 74 | 100 | 163 | 129 | 87 | 110 | 178 | 186 |
| 10 | NZR11 | 166 | 108 | 62 | 100 | 166 | 156 | 78 | 152 | 175 | 186 |
| 11 | NZR12 | 163 | 99 | 65 | 94 | 163 | 177 | 75 | 152 | 178 |  |
| 12 | NZR13 | 166 | 108 | 62 | 97 | 166 | 156 | 78 | 122 | 163 | 186 |
| 13 | NZR14 | 163 | 105 | 65 | 100 | 178 | 156 | 93 | 113 | 172 |  |
| 14 | NZR15 | 160 | 105 | 65 | 91 | 160 | 168 | 75 | 125 | 175 | 201 |
| 15 | NZR16 | 163 | 105 | 71 | 100 | 160 | 165 | 75 | 152 | 160 | 186 |
| 16 | NZR17 | 169 | 111 | 65 | 100 | 166 | 141 | 75 | 122 | 166 | 186 |
| 17 | NZR18 | 163 | 108 | 98 | 100 | 163 | 165 | 75 | 116 | 178 | 186 |
| 18 | NZR19 | 166 | 102 | 74 | 100 | 160 | 162 | 87 | 119 | 163 | 186 |
| 19 | NZR21 | 181 | 102 | 65 | 100 | 175 | 162 | 87 | 119 | 175 | 186 |
| 20 | NZR22 | 178 | 99 | 59 | 100 | 166 | 162 | 84 | 125 | 175 | 186 |
| 21 | NZR23 |  | 102 | 77 | 100 | 166 | 156 | 81 | 116 | 163 | 201 |
| 22 | NZR24 | 175 | 105 | 68 | 100 | 175 | 156 | 84 | 119 | 175 | 186 |
| 23 | NZR25 | 181 | 99 | 80 | 100 | 154 | 159 | 81 | 152 | 160 | 186 |
| 24 | NZR26 | 172 | 99 | 77 | 97 | 160 | 168 | 84 | 119 | 202 | 201 |
| 25 | NZR27 | 175 | 99 | 98 | 97 | 160 | 168 | 84 | 119 | 202 | 201 |
| 26 | NZR28 | 181 | 102 | 98 | 100 | 175 | 162 | 87 | 119 | 175 | 186 |
| 27 | NZR29 | 175 | 105 | 65 | 94 | 175 | 168 | 87 | 116 | 175 | 201 |
| 28 | NZR30 | 169 | 111 | 74 | 100 | 169 | 159 | 78 | 122 |  |  |
| 29 | NZR31 | 163 | 99 | 98 | 100 | 175 | 159 | 75 | 128 | 175 | 186 |
| 30 | NZR32 | 163 | 102 | 62 | 100 | 175 | 159 |  | 143 | 160 | 186 |
| 31 | NZR33 | 175 | 120 | 98 | 103 | 169 | 162 | 87 | 128 | 166 | 186 |
| 32 | NZR34 | 166 | 99 | 98 | 100 | 157 | 159 | 84 | 116 | 172 | 186 |
| 33 | NZR35 | 166 | 105 | 65 | 100 | 166 | 165 | 84 | 128 | 178 |  |
| 34 | NZR36 | 172 | 102 | 68 | 97 | 172 | 123 |  | 119 | 172 |  |
| 35 | NZR37 | 163 | 108 | 68 | 100 | 172 | 156 | 75 | 119 | 163 |  |
| 36 | NZR38 | 181 | 114 | 71 | 97 | 163 | 159 |  | 122 | 178 |  |
| 37 | NZR43 | 166 | 111 | 68 | 85 | 166 | 156 |  | 119 | 160 |  |
| 38 | NZR47 | 163 | 102 | 59 | 85 | 163 | 162 | 84 | 116 |  |  |
| 39 | NZR49 | 166 | 102 | 56 | 85 | 166 | 171 | 81 | 116 | 163 |  |
| 40 | NZR53 | 160 | 117 | 56 |  | 160 | 171 | 75 | 119 | 175 |  |
| 41 | NZR55 | 163 | 108 | 62 | 85 | 163 | 156 | 84 | 110 | 178 |  |
| 42 | NZR56 | 172 | 117 | 65 |  | 172 | 159 | 81 | 128 | 175 |  |
| 43 | NZR57 | 166 | 105 | 65 | 97 | 160 | 156 |  | 110 | 172 |  |
| 44 | NZR59 | 166 | 111 | 71 | 85 | 166 | 156 |  | 116 | 160 |  |
| 45 | BOK1 | 163 | 105 | 98 | 100 | 160 | 147 | 87 | 152 | 181 | 186 |
| 46 | BOK2 | 163 | 96 | 68 | 100 | 163 | 162 | 75 | 152 | 196 | 201 |
| 47 | BOK3 | 172 | 105 | 68 | 100 | 169 | 162 | 84 | 128 | 178 | 186 |
| 48 | BOK4 | 178 | 108 | 62 | 100 | 175 | 177 | 75 | 116 | 178 | 186 |
| 49 | BOK5 | 169 | 99 | 74 | 100 | 190 |  | 63 | 122 | 178 |  |
| 50 | BOK6 | 160 | 108 | 74 | 97 | 163 | 162 | 84 | 116 | 175 | 186 |
| 51 | BOK7 | 181 | 108 | 65 | 97 | 163 | 174 | 63 | 152 | 178 | 186 |
| 52 | BOK8 | 178 | 105 | 77 | 97 | 169 | 153 | 63 | 128 | 202 | 186 |
| 53 | BOK9 | 169 | 108 | 89 | 100 | 190 | 156 | 84 | 122 | 178 | 186 |
| 54 | BOK10 | 166 | 108 | 77 | 91 | 166 | 156 | 81 | 122 | 163 | 189 |
| 55 | BOK11 | 178 | 108 | 74 | 100 | 184 | 162 | 63 | 122 | 196 |  |
| 56 | BOK12 | 169 | 120 | 59 | 100 | 181 | 165 | 75 | 122 | 175 | 186 |
| 57 | BOK13 | 190 | 102 | 74 | 100 | 190 | 156 | 63 | 119 | 196 |  |
| 58 | BOK14 | 172 | 105 | 62 | 100 | 163 | 159 | 87 | 119 | 163 | 201 |
| 59 | BOK15 | 169 | 108 | 89 | 100 | 190 | 156 | 84 | 152 | 178 | 186 |
| 60 | BOK16 | 175 | 99 | 59 |  | 178 | 156 |  | 152 | 163 | 183 |
| 61 | BOK17 | 166 | 108 | 77 | 91 | 166 | 147 | 63 | 152 | 163 |  |
| 62 | BOK18 | 169 | 105 | 71 | 100 | 169 | 135 | 78 | 116 | 178 | 186 |
| 63 | BOK19 | 160 | 108 | 62 | 97 | 160 | 141 | 84 | 152 | 163 | 186 |
| 64 | BOK20 | 175 | 99 | 68 | 100 | 175 | 165 | 84 | 128 | 172 | 186 |
| 65 | BOK21 | 166 | 108 | 71 | 103 | 166 | 162 | 84 | 122 | 175 | 180 |
| 66 | BOK22 | 178 | 105 | 77 | 85 | 169 | 153 |  | 119 | 163 | 186 |
| 67 | BOK23 | 178 | 105 | 77 | 97 | 169 | 153 | 87 | 128 | 202 | 186 |
| 68 | BOK24 | 160 | 102 | 59 | 100 | 163 | 162 | 75 | 119 | 166 | 186 |
| 69 | BOK25 | 169 | 105 | 62 | 100 | 169 | 168 | 81 | 122 | 175 | 201 |
| 70 | BOK26 | 166 | 96 | 68 | 100 | 166 | 162 | 84 | 113 | 178 | 186 |
| 71 | BOK27 | 166 | 105 | 65 | 97 | 166 | 120 | 87 | 116 | 166 | 186 |
| 72 | BOK28 | 166 | 93 | 68 | 100 | 166 | 120 | 87 | 116 | 178 | 201 |
| 73 | BOK29 | 166 | 111 | 62 | 97 | 166 | 159 | 78 | 116 | 169 | 201 |
| 74 | BOK30 | 163 | 105 | 62 | 85 | 163 |  | 63 | 152 | 160 | 216 |
| 75 | BOK31 | 163 | 105 | 62 | 100 | 163 | 159 | 93 | 119 | 163 | 201 |
| 76 | BOK32 | 163 | 102 | 59 | 100 | 163 | 153 | 87 | 110 | 166 | 186 |
| 77 | BOK33 | 175 | 102 | 77 | 100 | 169 | 153 | 84 | 152 | 202 | 186 |
| 78 | FOR1 | 160 | 102 | 68 | 100 | 160 | 150 | 75 | 131 | 223 | 186 |
| 79 | FOR2 | 166 | 111 | 62 | 97 | 166 | 159 | 78 | 116 | 172 | 201 |
| 80 | FOR3 | 169 | 111 | 65 | 103 | 169 | 177 | 84 | 119 | 178 | 186 |
| 81 | FOR4 | 166 | 102 | 68 | 100 | 163 | 156 | 75 | 122 | 175 | 186 |
| 82 | FOR5 | 160 | 96 | 68 | 100 | 160 | 156 | 93 | 116 | 163 | 186 |
| 83 | FOR6 | 175 | 90 | 65 | 100 | 169 | 159 | 87 | 152 | 172 | 186 |
| 84 | FOR7 | 178 | 105 | 62 | 100 | 166 | 156 | 93 | 125 | 178 | 186 |
| 85 | FOR8 | 166 | 111 | 62 | 100 | 187 | 135 | 87 | 152 | 175 | 186 |
| 86 | FOR10 | 190 | 111 | 62 | 97 | 166 | 159 | 78 | 116 | 172 | 201 |
| 87 | BAS1 | 169 | 117 | 62 | 100 | 169 | 159 | 84 | 122 | 196 | 186 |
| 88 | BAS2 | 169 | 99 | 86 | 100 | 175 | 156 | 84 | 122 | 175 | 186 |
| 89 | BAS3 | 166 | 108 | 65 | 100 | 190 | 156 | 78 | 122 | 199 | 186 |
| 90 | BAS4 | 163 | 105 | 65 | 100 | 166 | 177 | 84 | 107 | 172 | 186 |
| 91 | BAS5 | 169 | 90 | 77 | 103 | 169 | 153 | 75 | 122 | 196 | 201 |
| 92 | BAS6 | 160 | 105 | 59 | 106 | 175 | 162 | 78 | 116 | 178 | 186 |
| 93 | BAS7 | 142 | 102 | 65 | 100 | 160 | 147 | 78 | 119 | 178 | 186 |
| 94 | BAS8 | 160 | 99 | 68 | 97 | 175 | 159 | 87 | 122 | 166 | 186 |
| 95 | BAS9 | 178 | 108 | 68 | 94 | 163 | 156 | 78 | 128 | 172 | 186 |
| 96 | BAS10 | 166 | 120 | 68 | 100 | 181 | 156 | 84 | 125 | 190 | 186 |
| 97 | BAS11 | 178 | 111 | 71 | 100 | 163 | 156 | 81 | 122 | 178 |  |
| 98 | BAS12 | 163 | 105 | 71 | 100 | 163 | 159 | 78 | 116 | 175 | 186 |
| 99 | BAS13 | 193 | 102 | 74 | 100 | 187 | 162 | 84 | 131 | 163 | 186 |
| 100 | BAS14 | 169 | 108 | 71 | 94 | 172 | 162 | 75 | 113 | 175 | 186 |
| 101 | BAS15 | 166 | 105 | 65 | 100 | 160 | 156 | 87 | 113 | 157 | 186 |
| 102 | BAS16 |  | 99 | 65 | 100 | 166 | 159 | 78 | 119 | 196 | 222 |
| 103 | BAS17 | 184 | 105 | 65 | 97 | 190 | 156 | 78 | 134 | 163 | 186 |
| 104 | BAS18 | 175 | 108 | 68 | 97 | 166 | 156 | 78 | 110 | 175 | 186 |
| 105 | BAS19 | 175 | 108 | 71 | 100 | 166 | 162 | 93 | 116 | 175 | 186 |
| 106 | BAS20 |  | 105 | 71 | 97 | 166 | 159 | 78 | 122 | 196 |  |
| 107 | BAS21 | 184 | 114 | 68 | 100 | 184 | 156 | 75 | 119 | 196 | 186 |
| 108 | BAS22 | 169 | 114 | 59 | 100 | 172 | 147 | 84 | 128 |  |  |
| 109 | BAS23 | 160 | 96 | 65 | 100 | 169 | 174 | 75 | 131 | 160 |  |
| 110 | BAS24 | 169 | 108 | 62 | 100 | 166 | 156 | 78 | 119 | 196 |  |
| 111 | BAS25 | 166 | 105 | 65 | 94 | 181 | 156 | 87 | 122 | 160 | 186 |
| 112 | BAS26 | 172 | 111 | 77 | 97 | 166 | 156 | 75 | 131 | 157 |  |
| 113 | BAS27 | 166 | 99 | 68 | 97 | 166 | 156 | 90 | 128 | 175 | 186 |
| 114 | BAS28 | 175 | 105 | 59 | 106 | 175 | 162 | 78 | 122 | 196 |  |
| 115 | BAS29 | 169 | 105 | 65 | 97 | 169 | 171 | 87 | 125 | 163 | 186 |
| 116 | BAS30 | 166 | 108 | 71 | 100 | 175 | 117 | 93 | 122 | 160 | 186 |
| 117 | BAS31 | 169 | 111 | 74 | 100 | 169 | 147 | 63 | 122 | 196 |  |
| 118 | BAS32 | 178 | 114 | 65 | 103 | 166 | 135 | 75 | 128 | 163 | 186 |
| 119 | BAS33 | 160 | 102 | 71 | 100 | 172 | 156 | 87 | 122 | 166 | 186 |
| 120 | CAI1 | 166 | 99 | 65 | 100 | 166 | 156 | 84 | 119 | 196 | 222 |
| 121 | CAI2 | 166 | 111 | 71 | 103 | 190 | 159 | 90 | 122 | 163 | 222 |
| 122 | CAI3 | 172 | 99 | 65 | 85 | 172 | 147 | 84 | 122 | 196 |  |
| 123 | CAI4 | 166 | 99 | 68 | 100 | 160 | 189 | 69 | 122 | 160 | 186 |
| 124 | CAI5 | 178 | 111 | 59 | 100 | 172 | 156 | 87 | 116 | 163 | 186 |
| 125 | CAI6 | 166 | 108 | 80 | 100 | 166 | 171 | 84 | 119 | 178 |  |
| 126 | CAI7 | 169 | 108 | 59 | 100 | 166 | 177 | 84 | 116 | 163 | 186 |
| 127 | CAI8 | 163 | 111 | 71 | 100 | 169 | 147 | 78 | 116 | 178 | 201 |
| 128 | CAI9 | 193 | 111 | 71 | 103 | 160 | 165 | 84 | 125 | 163 | 186 |
| 129 | CAI10 | 181 |  | 74 | 100 | 178 | 192 | 63 | 122 | 196 | 222 |
| 130 | CAI11 | 184 | 117 | 65 | 97 | 172 | 162 | 84 | 122 | 172 |  |
| 131 | CAI12 | 148 | 102 | 65 | 103 | 181 | 159 | 84 | 116 | 175 |  |
| 132 | FAR1 | 172 | 102 | 74 | 100 | 169 | 183 | 75 | 119 | 163 | 186 |
| 133 | FAR2 | 169 | 108 | 62 | 100 | 199 | 186 | 96 | 125 | 187 | 186 |
| 134 | FAR3 | 172 | 120 | 68 | 100 | 199 | 162 | 87 | 119 | 163 | 186 |
| 135 | FAR4 | 169 | 105 | 65 | 100 | 172 | 147 | 78 | 116 | 178 | 186 |
| 136 | FAR5 | 172 | 105 | 68 | 97 | 175 | 159 | 84 | 122 | 175 |  |
| 137 | FAR6 | 160 | 102 | 68 | 100 | 160 | 165 | 78 | 122 | 196 |  |
| 138 | FAR7 | 166 | 99 | 74 | 100 | 175 | 156 | 78 | 122 | 175 | 186 |
| 139 | FAR8 | 163 | 105 | 65 | 91 | 184 | 162 | 78 | 119 | 160 |  |
| 140 | FAR9 | 169 | 114 | 68 | 97 | 169 | 156 | 75 | 128 | 196 |  |
| 141 | FAR10 |  | 102 | 77 | 100 | 184 | 156 | 84 | 125 | 163 |  |
| 142 | FAR11 | 160 | 111 | 65 | 100 | 160 | 156 | 84 | 116 | 196 | 201 |
| 143 | FAR12 | 166 | 105 | 89 | 100 | 172 | 150 | 84 | 122 | 175 | 186 |
| 144 | FAR13 | 166 | 108 | 65 | 100 | 166 | 189 | 84 | 122 | 172 |  |
| 145 | FAR14 | 166 | 105 | 59 | 100 | 166 | 171 | 78 | 119 | 178 |  |
| 146 | FAR15 | 175 | 111 | 65 | 103 | 160 | 153 | 75 | 116 | 202 | 186 |
| 147 | FAR16 | 160 | 102 | 68 | 100 | 160 | 183 | 84 | 119 | 196 |  |
| 148 | FAR17 | 175 | 99 | 68 | 100 | 169 | 162 | 84 | 122 | 160 | 186 |
| 149 | FAR18 | 166 | 105 | 71 | 100 | 166 | 162 | 78 | 119 | 175 | 201 |
| 150 | FAR19 | 190 | 114 | 68 | 97 | 169 | 156 | 75 | 128 | 163 | 186 |
| 151 | FAR20 | 172 | 114 | 71 | 97 | 163 | 186 | 90 | 116 | 172 | 186 |
| 152 | FAR21 | 163 | 102 | 59 | 100 | 163 | 162 | 84 | 119 | 175 | 201 |
| 153 | FAR22 | 160 | 105 | 59 | 100 | 169 | 165 | 75 | 119 | 196 |  |
| 154 | FAR23 | 166 | 99 | 74 | 100 | 181 | 156 | 78 | 125 |  |  |
| 155 | FAR24 | 166 | 108 | 74 | 100 | 160 | 159 | 84 | 116 | 160 | 186 |
| 156 | FAR25 | 169 | 105 | 65 | 100 | 172 | 147 | 78 | 116 | 196 |  |
| 157 | FAR26 | 169 | 111 | 59 | 100 | 172 | 156 | 75 | 119 | 172 | 186 |
| 158 | FAR28 | 163 | 93 | 65 | 97 | 157 | 159 | 87 | 113 |  |  |
| 159 | FAR29 | 166 | 105 | 68 | 97 | 160 | 156 | 84 | 113 | 175 | 186 |
| 160 | FAR30 | 184 | 111 | 68 | 97 | 169 | 150 | 75 | 125 | 160 |  |
| 161 | FAR31 | 166 | 108 | 86 | 100 | 160 | 162 | 78 | 131 | 160 | 186 |
| 162 | FAR32 | 163 | 99 | 65 | 100 | 163 | 168 | 90 | 119 | 175 | 186 |
| 163 | FAR33 | 172 | 105 | 68 | 97 | 175 | 159 | 84 | 122 | 172 | 186 |
| 164 | FAR34 | 169 | 99 | 74 | 100 | 163 | 156 | 75 | 122 | 163 | 186 |
| 165 | FAR35 | 166 | 105 | 74 | 94 | 163 | 162 | 87 | 119 | 160 | 186 |
| 166 | FAR36 | 163 | 108 | 65 |  | 175 | 165 | 63 | 122 | 175 |  |
| 167 | FAR37 | 172 | 105 | 68 | 97 | 175 | 159 | 84 | 122 | 172 | 186 |
| 168 | FAR38 | 166 | 99 | 68 | 100 | 163 | 144 | 84 | 122 | 160 |  |
| 169 | FAR39 | 166 | 102 | 71 | 97 | 166 | 174 | 96 | 119 | 196 | 186 |
| 170 | FAR40 | 190 | 114 | 68 | 97 | 169 | 156 | 75 | 128 | 163 |  |
| 171 | FAR41 | 160 | 99 | 74 | 100 | 184 |  | 63 | 119 | 196 |  |
| 172 | FAR42 | 157 | 114 | 71 | 97 | 172 | 156 | 84 | 128 | 184 | 186 |
| 173 | FAR43 | 163 | 111 | 65 | 100 | 160 | 174 | 84 | 122 | 196 | 186 |
| 174 | GBA1 | 190 | 99 | 65 | 106 | 163 | 156 | 84 | 116 |  | 186 |
| 175 | GBA2 | 178 | 108 | 71 | 103 | 166 | 156 | 87 | 119 | 163 |  |
| 176 | GBA3 | 163 | 105 | 68 | 100 | 172 | 162 | 78 | 125 | 178 | 186 |
| 177 | GBA4 | 166 | 102 | 68 | 100 | 163 |  | 78 | 113 | 178 | 201 |
| 178 | GBA5 | 172 | 108 | 65 | 97 | 166 | 183 | 69 | 125 | 178 | 186 |
| 179 | GBA7 | 181 | 99 | 86 | 94 | 175 | 156 |  |  | 154 | 186 |
| 180 | GBA8 | 166 | 102 | 74 | 100 | 187 | 180 | 78 | 128 | 160 | 186 |
| 181 | GBA9 | 166 | 108 | 59 | 97 | 172 | 159 | 84 | 122 | 175 | 186 |
| 182 | GBA10 | 193 | 105 | 65 | 100 | 172 | 165 | 78 | 128 | 163 | 186 |
| 183 | GBA11 | 160 | 105 | 68 | 100 | 184 | 159 | 84 | 119 | 166 | 189 |
| 184 | GBA12 | 175 | 111 | 68 | 100 | 163 | 156 | 87 | 119 | 190 | 186 |
| 185 | GBA13 | 169 | 102 | 65 | 100 | 163 | 165 | 84 | 113 | 163 |  |
| 186 | GBA14 | 178 | 111 | 65 | 94 | 160 | 147 | 75 | 119 | 172 | 186 |
| 187 | GBA15 | 166 | 108 | 92 | 100 | 160 | 156 | 78 | 116 | 187 | 186 |
| 188 | GBA18 | 166 | 111 | 83 | 100 | 175 | 159 | 90 | 131 | 175 |  |
| 189 | GBA19 | 160 | 114 | 65 | 103 | 166 | 156 | 84 | 119 |  |  |
| 190 | GBA20 | 172 | 105 | 74 | 97 | 169 | 162 | 84 | 128 | 166 | 216 |
| 191 | GBA21 | 178 | 93 | 77 | 97 | 175 | 159 | 93 | 119 | 163 | 186 |
| 192 | GBA22 | 157 | 111 | 80 | 100 | 169 | 141 |  | 122 | 175 | 201 |
| 193 | GBA23 | 169 | 105 | 65 | 100 | 169 | 162 | 78 | 116 | 175 | 186 |
| 194 | GBA24 | 163 | 105 | 68 | 103 | 175 | 159 | 93 | 116 | 175 | 186 |
| 195 | GBA25 | 166 | 108 | 74 | 100 | 160 | 156 | 78 | 116 | 184 |  |
| 196 | GBA26 | 163 | 108 | 62 | 100 | 166 | 153 | 78 | 119 | 175 | 186 |
| 197 | GBA27 | 178 | 105 | 59 | 97 | 166 | 153 | 69 | 122 | 178 | 186 |
| 198 | GBA28 | 169 | 117 | 71 | 106 | 169 | 171 | 93 | 110 | 181 | 186 |
| 199 | GBA29 | 160 | 99 | 65 | 100 | 184 | 156 | 84 | 119 | 163 | 186 |
| 200 | GBA31 | 160 | 111 | 86 | 100 | 160 | 156 | 75 | 131 | 172 | 186 |
| 201 | GBA32 | 166 | 108 | 68 | 100 | 169 | 159 | 84 | 128 | 160 | 186 |
| 202 | GBA33 | 166 | 111 | 65 | 100 | 160 |  |  | 122 | 175 | 201 |
| 203 | GBA34 | 169 | 111 | 68 | 91 | 163 | 147 | 69 | 122 | 163 | 186 |
| 204 | GBA35 | 166 | 96 | 68 | 100 | 184 | 177 | 75 | 119 | 187 | 186 |
| 205 | GBA36 | 178 | 111 | 59 | 97 | 160 | 147 | 75 | 119 | 172 | 186 |
| 206 | GBA37 | 172 | 99 | 68 | 97 | 166 | 168 | 75 | 119 | 163 |  |
| 207 | GBA38 | 166 | 108 | 68 | 100 | 166 | 177 | 87 |  | 163 | 186 |
| 208 | GBA39 | 163 | 93 | 65 | 100 | 160 | 156 | 78 | 119 | 163 | 186 |
| 209 | GBA41 | 160 | 111 | 65 | 100 | 169 | 123 | 84 | 128 | 235 | 186 |
| 210 | GBA42 | 169 | 105 | 74 | 100 | 166 | 153 | 84 | 122 | 175 | 189 |
| 211 | GBA43 | 178 | 111 | 59 | 97 | 163 | 159 | 75 | 119 | 172 | 186 |
| 212 | GBA44 | 160 | 99 | 65 | 100 | 184 | 156 | 84 | 119 | 163 | 186 |
| 213 | GBA45 | 169 | 105 | 59 | 100 | 169 | 168 | 87 | 122 | 178 | 186 |
| 214 | GBA46 | 145 | 96 | 50 | 100 | 175 | 156 | 75 | 122 | 160 | 186 |
| 215 | GBA47 | 172 | 102 | 65 | 100 | 169 | 180 | 81 | 119 | 172 | 186 |
| 216 | GBA48 | 172 | 105 | 71 | 100 | 166 | 153 | 84 | 122 | 178 | 186 |
| 217 | GBA49 | 160 | 102 | 62 | 100 | 163 | 147 | 84 | 116 | 160 | 186 |
| 218 | GBA50 | 166 | 105 | 65 | 100 | 169 | 186 | 81 | 119 | 163 | 186 |
| 219 | GBA51 | 169 | 108 | 62 | 100 | 166 | 186 | 90 | 116 | 175 | 201 |
| 220 | GBA52 | 169 | 105 | 65 | 94 | 166 | 159 | 87 | 119 | 181 | 186 |
| 221 | GBA53 | 160 | 105 | 65 | 100 | 184 | 156 | 84 | 119 | 163 | 186 |
| 222 | GBA55 | 160 | 102 | 77 | 100 | 166 | 165 | 84 | 128 | 217 | 186 |
| 223 | GBA56 | 166 | 108 | 74 | 100 | 160 | 156 | 78 | 116 | 184 | 186 |
| 224 | GBA57 | 166 | 117 | 68 | 100 | 169 | 165 | 75 | 122 | 163 | 186 |
| 225 | GBA58 | 166 | 93 | 65 | 100 | 160 | 147 | 75 | 119 | 163 | 186 |
| 226 | GBA59 | 160 | 105 | 65 | 94 | 166 | 168 | 78 | 122 | 166 | 186 |
| 227 | GBA60 | 178 | 108 | 65 | 97 | 163 | 153 | 75 | 122 | 163 | 186 |
| 228 | GBA61 | 178 | 111 | 71 | 97 | 172 | 168 | 87 | 125 | 187 | 186 |
| 229 | GBA62 | 160 | 105 | 89 | 106 | 166 | 183 | 90 | 119 | 175 | 186 |
| 230 | GBA63 | 163 | 99 | 77 | 100 | 157 | 156 | 87 | 116 | 175 | 186 |
| 231 | GBA64 | 166 | 117 | 68 | 100 | 169 | 165 | 75 | 122 | 160 | 186 |
| 232 | GBA65 | 169 | 99 | 68 | 103 | 169 | 159 | 75 | 119 | 175 | 186 |
| 233 | GBA66 | 169 | 111 | 71 | 94 | 163 | 150 | 84 | 125 | 178 | 186 |
| 234 | GBA67 | 163 | 108 | 65 | 97 | 169 | 162 | 84 | 119 | 172 | 186 |
| 235 | GBA68 | 166 | 93 | 65 | 100 | 160 | 147 | 78 | 119 | 166 | 186 |
| 236 | GBA69 | 163 | 99 | 65 | 100 | 163 | 174 | 84 | 125 | 175 | 186 |
| 237 | GBA70 | 178 | 111 | 53 | 100 | 169 | 168 | 78 | 119 | 160 | 186 |
| 238 | GBA71 | 166 | 108 | 65 | 100 | 166 | 162 | 90 | 116 | 178 | 186 |
| 239 | GBA72 | 190 | 111 | 65 | 100 | 160 | 153 | 72 | 119 | 172 | 186 |
| 240 | GBA73 |  | 114 | 65 | 100 | 172 |  | 75 | 122 | 175 | 201 |
| 241 | GBA74 | 199 | 105 | 65 | 103 | 163 | 150 | 78 | 119 | 172 | 186 |
| 242 | GBA75 | 160 | 105 | 62 | 100 | 163 | 162 | 78 | 122 | 163 | 186 |
| 243 | GBA76 | 169 | 117 | 68 | 100 | 163 | 147 | 84 | 122 | 175 | 186 |
| 244 | GBA77 |  | 102 | 71 | 100 | 163 | 156 | 84 | 113 | 163 | 186 |
| 245 | GBA78 | 190 | 105 | 65 | 100 | 163 | 150 | 87 | 119 | 172 | 186 |
| 246 | GBA79 | 166 | 105 | 74 | 100 | 169 | 156 | 78 | 116 | 187 | 186 |
| 247 | GBA80 | 169 | 102 | 74 | 100 | 163 | 147 | 75 | 125 | 187 | 180 |
| 248 | GBA81 |  | 108 | 71 | 103 | 166 | 156 | 78 | 122 | 160 | 186 |
| 249 | GBA82 | 163 | 102 | 74 | 100 | 169 | 162 | 84 | 122 | 172 | 186 |
| 250 | GBA83 | 166 | 102 | 74 | 100 | 172 | 156 | 78 | 119 | 175 | 186 |
| 251 | GBA84 |  | 102 | 74 | 100 | 163 | 147 | 75 | 125 | 187 | 180 |
| 252 | GBA85 | 184 | 111 | 65 | 100 | 172 | 162 | 78 | 143 | 175 | 186 |
| 253 | SEN14 | 163 | 90 | 68 | 97 | 166 | 165 | 75 | 116 | 175 | 186 |
| 254 | SEN19 | 178 | 114 | 56 | 100 | 178 | 168 | 78 | 113 | 160 | 186 |
| 255 | SEN21 | 181 | 96 | 68 | 100 | 175 | 168 | 75 | 116 | 172 | 186 |
| 256 | SEN25 | 172 | 105 | 56 | 100 | 172 | 177 | 75 | 116 | 163 | 186 |
| 257 | SEN26 | 160 | 111 | 59 | 100 | 160 | 159 | 84 | 122 | 172 | 186 |
| 258 | SEN29 | 187 | 99 | 68 | 97 | 187 | 168 | 75 | 116 | 163 | 186 |
| 259 | SEN30 | 163 | 111 | 59 | 112 | 160 | 159 | 84 | 122 | 175 |  |
| 260 | SEN32 | 163 | 105 | 74 | 103 | 163 | 162 | 84 | 119 | 175 | 186 |
| 261 | SEN38 | 184 | 99 | 68 | 97 | 169 | 156 | 87 | 119 | 172 |  |
| 262 | SEN39 | 184 | 99 | 56 | 97 | 184 | 156 |  | 122 | 172 |  |
| 263 | SEN42 | 184 | 99 | 68 | 97 | 184 | 156 |  | 122 |  |  |
| 264 | SEN44 | 169 | 96 | 56 | 103 | 169 | 153 |  | 122 |  |  |
| 265 | SEN45 | 160 | 99 | 68 | 100 | 172 | 156 | 87 | 113 | 163 |  |
| 266 | SEN46 | 184 | 99 | 68 | 97 | 169 | 156 |  | 119 |  |  |
| 267 | SEN56 | 175 | 99 | 68 | 97 | 187 | 168 | 75 | 116 | 166 | 186 |
| 268 | SEN58 |  | 99 | 77 | 100 | 184 | 192 |  | 122 |  |  |

Highlighted light blue predominant alleles within mixed genotype infections while single unmixed alleles are not highlighted. Missing genotype data is reflected by gaps.
