# Supplementary material for: Population genetic structure of Plasmodium falciparum across a region of diverse endemicity in West Africa
Source: Malar J. 2012 Jul 3;11:223. doi: 10.1186/1475-2875-11-223 (PMC3425276; doi:10.1186/1475-2875-11-223)
Supplement: Additional file 2 — Table S2. Allele frequencies at 10 microsatellite loci in each of the eight population samples ofPlasmodium falciparum. Data on frequency of each allele scored for each microsatellite locus in each of the eight populations sampled. [file 1475-2875-11-223-S2.docx]

**Table S2.** Allele frequencies at 10 microsatellite loci in each of the eight population samples of *Plasmodium falciparum*

| Locus | Allele | Nzr | Bok | For | Bas | Cai | Far | Gba | Ric |
| --- | --- | --- | --- | --- | --- | --- | --- | --- | --- |
| TA1 | 142 | 0 | 0 | 0 | 0.032 | 0 | 0 | 0 | 0 |
|  | 145 | 0 | 0 | 0 | 0 | 0 | 0 | 0.013 | 0 |
|  | 148 | 0 | 0 | 0 | 0 | 0.083 | 0 | 0 | 0 |
|  | 157 | 0 | 0 | 0 | 0 | 0 | 0.024 | 0.013 | 0 |
|  | 160 | 0.093 | 0.091 | 0.222 | 0.129 | 0 | 0.122 | 0.16 | 0.133 |
|  | 163 | 0.256 | 0.152 | 0 | 0.065 | 0.083 | 0.146 | 0.107 | 0.2 |
|  | 166 | 0.256 | 0.212 | 0.333 | 0.194 | 0.333 | 0.293 | 0.253 | 0 |
|  | 169 | 0.093 | 0.182 | 0.111 | 0.258 | 0.083 | 0.146 | 0.16 | 0.067 |
|  | 172 | 0.093 | 0.061 | 0 | 0.032 | 0.083 | 0.146 | 0.067 | 0.067 |
|  | 175 | 0.093 | 0.091 | 0.111 | 0.097 | 0 | 0.049 | 0.013 | 0.067 |
|  | 178 | 0.023 | 0.152 | 0.111 | 0.097 | 0.083 | 0 | 0.12 | 0.067 |
|  | 181 | 0.093 | 0.03 | 0 | 0 | 0.083 | 0 | 0.013 | 0.067 |
|  | 184 | 0 | 0 | 0 | 0.065 | 0.083 | 0.024 | 0.013 | 0.267 |
|  | 187 | 0 | 0 | 0 | 0 | 0 | 0 | 0 | 0.067 |
|  | 190 | 0 | 0.03 | 0.111 | 0 | 0 | 0.049 | 0.04 | 0 |
|  | 193 | 0 | 0 | 0 | 0.032 | 0.083 | 0 | 0.013 | 0 |
|  | 199 | 0 | 0 | 0 | 0 | 0 | 0 | 0.013 | 0 |
|  |  |  |  |  |  |  |  |  |  |
|  | n | 43 | 33 | 9 | 31 | 12 | 41 | 75 | 15 |
|  |  |  |  |  |  |  |  |  |  |
|  |  |  |  |  |  |  |  |  |  |
| TAA87 | 90 | 0 | 0 | 0.111 | 0.03 | 0 | 0 | 0 | 0.063 |
|  | 93 | 0 | 0.03 | 0 | 0 | 0 | 0.024 | 0.051 | 0 |
|  | 96 | 0.045 | 0.061 | 0.111 | 0.03 | 0 | 0 | 0.025 | 0.125 |
|  | 99 | 0.205 | 0.091 | 0 | 0.121 | 0.273 | 0.167 | 0.101 | 0.5 |
|  | 102 | 0.227 | 0.121 | 0.222 | 0.091 | 0.091 | 0.143 | 0.139 | 0 |
|  | 105 | 0.227 | 0.333 | 0.111 | 0.273 | 0 | 0.286 | 0.241 | 0.125 |
|  | 108 | 0.114 | 0.303 | 0 | 0.212 | 0.182 | 0.119 | 0.177 | 0 |
|  | 111 | 0.091 | 0.03 | 0.444 | 0.091 | 0.364 | 0.119 | 0.19 | 0.125 |
|  | 114 | 0.023 | 0 | 0 | 0.091 | 0 | 0.119 | 0.025 | 0.063 |
|  | 117 | 0.045 | 0 | 0 | 0.03 | 0.091 | 0 | 0.051 | 0 |
|  | 120 | 0.023 | 0.03 | 0 | 0.03 | 0 | 0.024 | 0 | 0 |
|  |  |  |  |  |  |  |  |  |  |
|  | n | 44 | 33 | 9 | 33 | 11 | 42 | 79 | 16 |
|  |  |  |  |  |  |  |  |  |  |
|  |  |  |  |  |  |  |  |  |  |
| ARA2 | 50 | 0 | 0 | 0 | 0 | 0 | 0 | 0.013 | 0 |
|  | 53 | 0 | 0 | 0 | 0 | 0 | 0 | 0.013 | 0 |
|  | 56 | 0.045 | 0 | 0 | 0 | 0 | 0 | 0 | 0.25 |
|  | 59 | 0.045 | 0.121 | 0 | 0.091 | 0.167 | 0.095 | 0.063 | 0.125 |
|  | 62 | 0.114 | 0.212 | 0.444 | 0.061 | 0 | 0.024 | 0.051 | 0 |
|  | 65 | 0.227 | 0.061 | 0.222 | 0.303 | 0.333 | 0.238 | 0.354 | 0 |
|  | 68 | 0.136 | 0.152 | 0.333 | 0.182 | 0.083 | 0.31 | 0.177 | 0.5 |
|  | 71 | 0.068 | 0.061 | 0 | 0.212 | 0.25 | 0.095 | 0.089 | 0 |
|  | 74 | 0.091 | 0.121 | 0 | 0.061 | 0.083 | 0.167 | 0.127 | 0.063 |
|  | 77 | 0.045 | 0.182 | 0 | 0.061 | 0 | 0.024 | 0.038 | 0.063 |
|  | 80 | 0.023 | 0 | 0 | 0 | 0.083 | 0 | 0.013 | 0 |
|  | 83 | 0 | 0 | 0 | 0 | 0 | 0 | 0.013 | 0 |
|  | 86 | 0 | 0 | 0 | 0.03 | 0 | 0.024 | 0.025 | 0 |
|  | 89 | 0 | 0.061 | 0 | 0 | 0 | 0.024 | 0.013 | 0 |
|  | 92 | 0 | 0 | 0 | 0 | 0 | 0 | 0.013 | 0 |
|  | 95 | 0.023 | 0 | 0 | 0 | 0 | 0 | 0 | 0 |
|  | 98 | 0.182 | 0.03 | 0 | 0 | 0 | 0 | 0 | 0 |
|  |  |  |  |  |  |  |  |  |  |
|  | n | 44 | 33 | 9 | 33 | 12 | 42 | 79 | 16 |
|  |  |  |  |  |  |  |  |  |  |
|  |  |  |  |  |  |  |  |  |  |
| PF377 | 85 | 0.119 | 0.063 | 0 | 0 | 0.083 | 0 | 0 | 0 |
|  | 91 | 0.024 | 0.063 | 0 | 0 | 0 | 0.024 | 0.013 | 0 |
|  | 94 | 0.095 | 0 | 0 | 0.091 | 0 | 0.024 | 0.063 | 0 |
|  | 97 | 0.167 | 0.219 | 0.222 | 0.212 | 0.083 | 0.293 | 0.139 | 0.438 |
|  | 100 | 0.571 | 0.625 | 0.667 | 0.576 | 0.583 | 0.634 | 0.671 | 0.375 |
|  | 103 | 0.024 | 0.031 | 0.111 | 0.061 | 0.25 | 0.024 | 0.076 | 0.125 |
|  | 106 | 0 | 0 | 0 | 0.061 | 0 | 0 | 0.038 | 0 |
|  | 112 | 0 | 0 | 0 | 0 | 0 | 0 | 0 | 0.063 |
|  |  |  |  |  |  |  |  |  |  |
|  | n | 42 | 32 | 9 | 33 | 12 | 41 | 79 | 16 |
|  |  |  |  |  |  |  |  |  |  |
|  |  |  |  |  |  |  |  |  |  |
| PFPK2 | 154 | 0.023 | 0 | 0 | 0 | 0 | 0 | 0 | 0 |
|  | 157 | 0.045 | 0 | 0 | 0 | 0 | 0.024 | 0.013 | 0 |
|  | 160 | 0.205 | 0.061 | 0.222 | 0.061 | 0.167 | 0.19 | 0.139 | 0.125 |
|  | 163 | 0.182 | 0.242 | 0.111 | 0.091 | 0 | 0.143 | 0.215 | 0.063 |
|  | 166 | 0.205 | 0.212 | 0.333 | 0.273 | 0.25 | 0.095 | 0.203 | 0.063 |
|  | 169 | 0.091 | 0.212 | 0.222 | 0.152 | 0.083 | 0.167 | 0.203 | 0.188 |
|  | 172 | 0.068 | 0 | 0 | 0.091 | 0.25 | 0.119 | 0.089 | 0.125 |
|  | 175 | 0.136 | 0.061 | 0 | 0.152 | 0 | 0.119 | 0.063 | 0.063 |
|  | 178 | 0.023 | 0.03 | 0 | 0 | 0.083 | 0 | 0 | 0.063 |
|  | 181 | 0.023 | 0.03 | 0 | 0.061 | 0.083 | 0.024 | 0 | 0 |
|  | 184 | 0 | 0.03 | 0 | 0.03 | 0 | 0.071 | 0.063 | 0.188 |
|  | 187 | 0 | 0 | 0.111 | 0.03 | 0 | 0 | 0.013 | 0.125 |
|  | 190 | 0 | 0.121 | 0 | 0.061 | 0.083 | 0 | 0 | 0 |
|  | 199 | 0 | 0 | 0 | 0 | 0 | 0.048 | 0 | 0 |
|  |  |  |  |  |  |  |  |  |  |
|  | n | 44 | 33 | 9 | 33 | 12 | 42 | 79 | 16 |
|  |  |  |  |  |  |  |  |  |  |
|  |  |  |  |  |  |  |  |  |  |
| Polya | 117 | 0 | 0 | 0 | 0.03 | 0 | 0 | 0 | 0 |
|  | 120 | 0 | 0.065 | 0 | 0 | 0 | 0 | 0 | 0 |
|  | 123 | 0.023 | 0 | 0 | 0 | 0 | 0 | 0.013 | 0 |
|  | 129 | 0.023 | 0 | 0 | 0 | 0 | 0 | 0 | 0 |
|  | 135 | 0 | 0.032 | 0.111 | 0.03 | 0 | 0 | 0 | 0 |
|  | 138 | 0.023 | 0 | 0 | 0 | 0 | 0 | 0 | 0 |
|  | 141 | 0.023 | 0.032 | 0 | 0 | 0 | 0 | 0.013 | 0 |
|  | 144 | 0 | 0 | 0 | 0 | 0 | 0.024 | 0 | 0 |
|  | 147 | 0 | 0.065 | 0 | 0.091 | 0.167 | 0.049 | 0.118 | 0 |
|  | 150 | 0 | 0 | 0.111 | 0 | 0 | 0.049 | 0.039 | 0 |
|  | 153 | 0 | 0.161 | 0 | 0.03 | 0 | 0.024 | 0.079 | 0.063 |
|  | 156 | 0.273 | 0.161 | 0.333 | 0.424 | 0.167 | 0.268 | 0.25 | 0.313 |
|  | 159 | 0.205 | 0.097 | 0.333 | 0.152 | 0.167 | 0.122 | 0.118 | 0.125 |
|  | 162 | 0.159 | 0.226 | 0 | 0.152 | 0.083 | 0.171 | 0.105 | 0.063 |
|  | 165 | 0.068 | 0.065 | 0 | 0 | 0.083 | 0.073 | 0.066 | 0.063 |
|  | 168 | 0.136 | 0.032 | 0 | 0 | 0 | 0.024 | 0.066 | 0.25 |
|  | 171 | 0.045 | 0 | 0 | 0.03 | 0.083 | 0.024 | 0.013 | 0 |
|  | 174 | 0 | 0.032 | 0 | 0.03 | 0 | 0.049 | 0.013 | 0 |
|  | 177 | 0.023 | 0.032 | 0.111 | 0.03 | 0.083 | 0 | 0.026 | 0.063 |
|  | 180 | 0 | 0 | 0 | 0 | 0 | 0 | 0.026 | 0 |
|  | 183 | 0 | 0 | 0 | 0 | 0 | 0.049 | 0.026 | 0 |
|  | 186 | 0 | 0 | 0 | 0 | 0 | 0.049 | 0.026 | 0 |
|  | 189 | 0 | 0 | 0 | 0 | 0.083 | 0.024 | 0 | 0 |
|  | 192 | 0 | 0 | 0 | 0 | 0.083 | 0 | 0 | 0.063 |
|  |  |  |  |  |  |  |  |  |  |
|  | n | 44 | 31 | 9 | 33 | 12 | 41 | 76 | 16 |
|  |  |  |  |  |  |  |  |  |  |
|  |  |  |  |  |  |  |  |  |  |
| TAA60 | 63 | 0 | 0.226 | 0 | 0.03 | 0.083 | 0.048 | 0 | 0 |
|  | 69 | 0 | 0 | 0 | 0 | 0.083 | 0 | 0.039 | 0 |
|  | 72 | 0 | 0 | 0 | 0 | 0 | 0 | 0.013 | 0 |
|  | 75 | 0.257 | 0.129 | 0.222 | 0.182 | 0 | 0.214 | 0.197 | 0.455 |
|  | 78 | 0.086 | 0.065 | 0.222 | 0.333 | 0.083 | 0.214 | 0.25 | 0.091 |
|  | 81 | 0.114 | 0.065 | 0 | 0.03 | 0 | 0 | 0.026 | 0 |
|  | 84 | 0.314 | 0.29 | 0.111 | 0.182 | 0.583 | 0.357 | 0.276 | 0.273 |
|  | 87 | 0.2 | 0.194 | 0.222 | 0.152 | 0.083 | 0.071 | 0.105 | 0.182 |
|  | 90 | 0 | 0 | 0 | 0.03 | 0.083 | 0.048 | 0.053 | 0 |
|  | 93 | 0.029 | 0.032 | 0.222 | 0.061 | 0 | 0 | 0.039 | 0 |
|  | 96 | 0 | 0 | 0 | 0 | 0 | 0.048 | 0 | 0 |
|  |  |  |  |  |  |  |  |  |  |
|  | n | 35 | 31 | 9 | 33 | 12 | 42 | 76 | 11 |
|  |  |  |  |  |  |  |  |  |  |
|  |  |  |  |  |  |  |  |  |  |
| TAA81 | 107 | 0 | 0 | 0 | 0.03 | 0 | 0 | 0 | 0 |
|  | 110 | 0.091 | 0.03 | 0 | 0.03 | 0 | 0 | 0.013 | 0 |
|  | 113 | 0.023 | 0.03 | 0 | 0.061 | 0 | 0.048 | 0.039 | 0.125 |
|  | 116 | 0.205 | 0.182 | 0.333 | 0.091 | 0.333 | 0.143 | 0.143 | 0.313 |
|  | 119 | 0.273 | 0.152 | 0.111 | 0.121 | 0.167 | 0.31 | 0.364 | 0.188 |
|  | 122 | 0.114 | 0.212 | 0.111 | 0.364 | 0.417 | 0.286 | 0.234 | 0.375 |
|  | 125 | 0.045 | 0 | 0.111 | 0.061 | 0.083 | 0.095 | 0.091 | 0 |
|  | 128 | 0.091 | 0.121 | 0 | 0.121 | 0 | 0.095 | 0.078 | 0 |
|  | 131 | 0 | 0 | 0.111 | 0.091 | 0 | 0.024 | 0.026 | 0 |
|  | 134 | 0 | 0 | 0 | 0.03 | 0 | 0 | 0 | 0 |
|  | 143 | 0.023 | 0 | 0 | 0 | 0 | 0 | 0.013 | 0 |
|  | 152 | 0.136 | 0.273 | 0.222 | 0 | 0 | 0 | 0 | 0 |
|  |  |  |  |  |  |  |  |  |  |
|  | n | 44 | 33 | 9 | 33 | 12 | 42 | 77 | 16 |
|  |  |  |  |  |  |  |  |  |  |
|  |  |  |  |  |  |  |  |  |  |
| TAA109 | 154 | 0 | 0 | 0 | 0 | 0 | 0 | 0.013 | 0 |
|  | 157 | 0 | 0 | 0 | 0.063 | 0 | 0 | 0 | 0 |
|  | 160 | 0.122 | 0.03 | 0 | 0.094 | 0.083 | 0.175 | 0.091 | 0.083 |
|  | 163 | 0.22 | 0.212 | 0.111 | 0.125 | 0.333 | 0.15 | 0.221 | 0.25 |
|  | 166 | 0.049 | 0.091 | 0 | 0.063 | 0 | 0 | 0.052 | 0.083 |
|  | 169 | 0 | 0.03 | 0 | 0 | 0 | 0 | 0 | 0 |
|  | 172 | 0.098 | 0.03 | 0.333 | 0.063 | 0.083 | 0.125 | 0.13 | 0.333 |
|  | 175 | 0.268 | 0.121 | 0.222 | 0.188 | 0.083 | 0.2 | 0.221 | 0.25 |
|  | 178 | 0.195 | 0.273 | 0.222 | 0.094 | 0.167 | 0.05 | 0.104 | 0 |
|  | 181 | 0 | 0.03 | 0 | 0 | 0 | 0 | 0.026 | 0 |
|  | 184 | 0 | 0 | 0 | 0 | 0 | 0.025 | 0.026 | 0 |
|  | 187 | 0 | 0 | 0 | 0 | 0 | 0.025 | 0.078 | 0 |
|  | 190 | 0 | 0 | 0 | 0.031 | 0 | 0 | 0.013 | 0 |
|  | 196 | 0 | 0.091 | 0 | 0.25 | 0.25 | 0.225 | 0 | 0 |
|  | 199 | 0 | 0 | 0 | 0.031 | 0 | 0 | 0 | 0 |
|  | 202 | 0.049 | 0.091 | 0 | 0 | 0 | 0.025 | 0 | 0 |
|  | 217 | 0 | 0 | 0 | 0 | 0 | 0 | 0.013 | 0 |
|  | 223 | 0 | 0 | 0.111 | 0 | 0 | 0 | 0 | 0 |
|  | 235 | 0 | 0 | 0 | 0 | 0 | 0 | 0.013 | 0 |
|  |  |  |  |  |  |  |  |  |  |
|  | n | 41 | 33 | 9 | 32 | 12 | 40 | 77 | 12 |
|  |  |  |  |  |  |  |  |  |  |
|  |  |  |  |  |  |  |  |  |  |
| TA42 | 180 | 0 | 0.034 | 0 | 0 | 0 | 0 | 0.027 | 0 |
|  | 183 | 0 | 0.034 | 0 | 0 | 0 | 0 | 0 | 0 |
|  | 186 | 0.793 | 0.655 | 0.778 | 0.92 | 0.5 | 0.88 | 0.863 | 1 |
|  | 189 | 0 | 0.034 | 0 | 0 | 0 | 0 | 0.027 | 0 |
|  | 201 | 0.207 | 0.207 | 0.222 | 0.04 | 0.125 | 0.12 | 0.068 | 0 |
|  | 216 | 0 | 0.034 | 0 | 0 | 0 | 0 | 0.014 | 0 |
|  | 222 | 0 | 0 | 0 | 0.04 | 0.375 | 0 | 0 | 0 |
|  |  |  |  |  |  |  |  |  |  |
|  | n | 29 | 29 | 9 | 25 | 8 | 25 | 73 | 8 |
|  |  |  |  |  |  |  |  |  |  |

Nzr = N’Zerekore; Bok = Boke; For = Forecariah; Bas = Basse; Cai = Caio; Far = Farafenni; Gba = Greater Banjul area; Ric = Richard Toll
